# Patterns of electronic and traditional cigarette use among college students: a multicenter study using network analysis

**DOI:** 10.1192/j.eurpsy.2026.10847

**Published:** 2026-07-31

**Authors:** A. L. Monezi Andrade, F. A. Pereira, H. S. Kim, L. S. da Silva, L. B. Nucci, W. A. de Oliveira

**Affiliations:** 1 School of Life Sciences, Pontifical Catholic University of Campinas, Campinas, Brazil; 2 Departament of Psychology, Toronto Metropolitan University, Toronto, Canada

## Abstract

**Introduction:**

The use of electronic (EC) and traditional (TC) cigarettes has been increasing among young adults, creating distinct profiles, including dual use (EC+TC). These patterns are associated with emotional vulnerability, impulsivity, and a lower quality of life; however, little is known about their interactions at the network level.

**Objectives:**

To determine the prevalence of nonsmokers, exclusive EC users, TC users, and dual users; to compare these groups on emotional indicators, alcohol consumption, and quality of life; and to use network analysis to identify key connections.

**Methods:**

This multicenter, cross-sectional study of 1,480 college students from Brazil and Canada. Participants completed a sociodemographic questionnaire along with standardized tools: DASS-21 (for depression, anxiety, and stress), DERS-18 (for emotional dysregulation), short UPPS-P (for impulsivity), WHOQOL-Bref (for quality of life), and assessments of alcohol use (AUDIT, doses, and binge drinking). Smoking status was categorized as EC and TC. Descriptive analyses, chi-squared tests, ANOVA, and network analysis using the EBICglasso algorithm were conducted, including the estimation of weighted partial correlations and evaluation of centrality indices. The study received approval from the ethics committees in both Brazil and Canada.

**Results:**

The sample comprised 63.4% nonsmokers and 36.6% smokers, including 37.3% exclusive EC smokers, 15.0% TC smokers, and 47.7% dual users. Smokers showed higher levels of emotional symptoms, dysregulation, and alcohol use, along with lower quality of life (p<0.05). Dual users had the worst profiles. The networks indicated higher density among smokers, especially dual users, with anxiety, stress, and binge drinking as key high-centrality nodes.

**Conclusions:**

Cigarette use, especially dual smoking, is linked to increased emotional vulnerability and poorer health indicators. Network analysis identified anxiety, stress, and binge drinking as priority targets for intervention, emphasizing the value of multicenter studies and network models in guiding preventive strategies for university students.

**Disclosure of Interest:**

None Declared

